# Detection of Tip-Burn Stress on Lettuce Grown in an Indoor Environment Using Deep Learning Algorithms

**DOI:** 10.3390/s22197251

**Published:** 2022-09-24

**Authors:** Munirah Hayati Hamidon, Tofael Ahamed

**Affiliations:** 1 Graduate School of Science and Technology, University of Tsukuba, 1-1-1 Tennodai, Tsukuba 305-8577, Japan; 2 Faculty of Life and Environmental Sciences, University of Tsukuba, 1-1-1 Tennodai, Tsukuba 305-8577, Japan

**Keywords:** tip-burn, lettuce, indoor farming, deep learning, YOLO, CenterNet

## Abstract

Lettuce grown in indoor farms under fully artificial light is susceptible to a physiological disorder known as tip-burn. A vital factor that controls plant growth in indoor farms is the ability to adjust the growing environment to promote faster crop growth. However, this rapid growth process exacerbates the tip-burn problem, especially for lettuce. This paper presents an automated detection of tip-burn lettuce grown indoors using a deep-learning algorithm based on a one-stage object detector. The tip-burn lettuce images were captured under various light and indoor background conditions (under white, red, and blue LEDs). After augmentation, a total of 2333 images were generated and used for training using three different one-stage detectors, namely, CenterNet, YOLOv4, and YOLOv5. In the training dataset, all the models exhibited a mean average precision (mAP) greater than 80% except for YOLOv4. The most accurate model for detecting tip-burns was YOLOv5, which had the highest mAP of 82.8%. The performance of the trained models was also evaluated on the images taken under different indoor farm light settings, including white, red, and blue LEDs. Again, YOLOv5 was significantly better than CenterNet and YOLOv4. Therefore, detecting tip-burn on lettuce grown in indoor farms under different lighting conditions can be recognized by using deep-learning algorithms with a reliable overall accuracy. Early detection of tip-burn can help growers readjust the lighting and controlled environment parameters to increase the freshness of lettuce grown in plant factories.

## 1. Introduction

Indoor vertical farms have been developed to grow fresh high-quality vegetables in buildings without being restricted by extreme climate or land availability limitations. Indoor farms serve as a significant alternative or supplement to conventional agriculture to meet the demands of major cities seeking fresh, safe, and locally grown veggies. To promote faster crop growth and to maximize total production within limited cultivation areas, the plants grown on indoor farms are highly dependent on artificial light sources [1]. Lettuce is one of the most widely planted vegetables grown in indoor farms, not only because of its nutritional content, but also due to its short growth cycle and high planting density. However, lettuce grown with this rapid growth process is prone to a physiological disorder known as tip-burn. Tip-burn is a major problem for most vegetable cultivation under a controlled environment [2], especially the completely closed environment of an indoor farm equipped with artificial light [3,4].

The primary cause of tip-burn stress which occurs on plants cultivated in completely closed environments, such as indoor farms, is calcium deficiency [5,6]. The deficiency is not because of a lack of calcium in the supply nutrients but rather is caused by the inability of calcium to enter the rapidly developing younger leaves. Commonly, the deficiency symptoms appear first on these younger leaves as calcium is one of the immobile nutrients that helps in leaf formation and growth. Due to the rapid growth changes, the leaf grows faster than the supply of calcium reaching the growth areas, which limits the plant’s ability to translocate an appropriate amount of calcium to a specific portion of the leaves. Additionally, environmental conditions, including a poorly formed root system, high light intensity, high electrical conductivity (EC), insufficient air movement, especially between the plants, and fluctuating temperatures and humidity also contribute to the incidence of tip-burn [1,2,5].

The typical symptom of tip-burn is when necrotic (brown) spots can be seen on the tips and margins of the rapidly developing young leaves of the lettuce. The affected leaves deform and cannot grow properly as they expand, which reduces the quality of the lettuce and significantly affects its commercial value [1,2,3]. Therefore, the detection of tip-burns in an early stage is crucial so that a proper and timely treatment process can be performed. Currently, a visual assessment is the primary method used by experts and growers to identify tip-burn problems [7]. This method may often result in poor decisions because it is highly prone to human error, which will negatively impact agricultural products. Furthermore, particularly under the complicated and condensed growth conditions of an indoor farming system, the availability of specialists in assessing such issues may be limited and difficult. Thus, using computer or machine vision systems to automatically identify tip-burn problems is the most impactful method for early and proper treatment processes to maintain leaf quality. 

Several machine vision applications have been developed to identify plant diseases or stress, such as the imaging method based on visible and near-infrared reflectance [8]. These methods combine spectral information with machine vision information. However, a detection process based on reflectance is not suitable for plant growth in dense conditions inside indoor environments due to the difficulty in image acquisition, environment constraint, and accessibility [8]. Additionally, a high implementation cost, time-consuming process, and the requirement for laboratory equipment setup make it ineffective for automatic and real-time identification of plant diseases [9]. In the training or learning process of image classification or object detection, deep-learning-based convolutional neural network has the potential for high accuracy of prediction with minimum preprocessing [8,10].

Deep neural network models can be implemented in the plant factories for near real-time detection of tip-burn. The evaluation of these models may provide new insight related to tip-burn detection, particularly in choosing the best deep-learning model for the relevant task. Furthermore, having an automated tip-burn detection for indoor farming or plant factories can alert growers to manipulate the controlled growing environment or regulate the nutrient supply system by increasing the calcium concentration, especially in inner leaves. Different indoor farming systems may adopt different light conditions, as they impact plant growth, physiology, and quality [11]. There are different spectrums of lighting that is also required for plant growth in the indoor plant environment. Thus, it is highly necessary to detect tip-burn at the different lighting conditions for prompt treatment of leaves and to provide nutrients so that they reach the deficient areas. 

Therefore, the purpose of this research is to detect tip-burn disease of lettuce from single images captured under different light conditions (colors) in an indoor plant growing system. The light condition and background of the images are required to adjust with indoor plant growth environment under white, red, and blue LED colors. To achieve the purpose of this study, images were collected from the different lighting conditions and the images were trained with three different established one-stage detection models: CenterNet, YOLOv4, and YOLOv5. In this regard, this paper is organized into several sections: Section 1 highlights the tip-burn disease problem of indoor systems. In Section 2, relevant works and the potential of deep learning are discussed. Section 3 presents the dataset used for training and testing to predict tip-burn in lettuce plants. Section 4 and Section 5 discuss the results of applying deep-learning models for tip-burn detection. Finally, Section 6 summarizes this work by presenting concluding remarks on the results and future research.

## 2. Related Works

A convolutional neural network (CNN) is a subset of the deep-learning techniques that are frequently used on image data to perform a variety of tasks, such as segmentation, object detection, and image classification. Generally, deep-learning-based detection algorithms can be divided into two-stage object detection and one-stage object detection. The core principle of a two-stage object detection is based on the use of a selective search algorithm to create a region proposal in the image for the targeted object, which is subsequently classified using a CNN [12]. Some popular models of this method include SPP-Net [13], RCNN [14], Fast R-CNN [15], and Faster R-CNN [16]. High detection accuracy may be attained with these approaches, but the drawbacks are the complexity of the network that require a longer training time and result in a diminished detection speed. In contrast, a one-stage detection method predicts all the bounding boxes in only a single run through the neural network [12]. Examples of the one-stage detector are SSD [17], YOLO [18], and CenterNet [19]. These one-stage detectors are not only able to reach high accuracies, but also have a faster processing speed [12], making them notable in the field of agriculture, where plant images are collected and utilized to classify plant species [20,21,22], count plants or fruits [23,24], identify pests [25,26,27] and weeds [28,29], and detect diseases [26,27,30,31,32,33,34]. 

Despite the fact that there are many deep-learning studies on the detection of different plant diseases, most of them have focused on plant detection in outdoor environments. Several public datasets have greatly contributed to plant disease detection, such as PlantVillage [20], PlantDoc [30], and PlantLeaves [35]. However, all the publicly accessible datasets only have available images of plant diseases that were cultivated in an outdoor environment. No public datasets are found for diseases or for stress analyses in indoor environments, particularly in indoor farming or plant factories, where plants are cultivated in multilayer structures that are closely clustered and are under artificial light [36,37]. 

Early detection of tip-burns in lettuce in the highly dense growing conditions of indoor environments is of great importance in reducing the cost of manual identification and improving lettuce quality and yield. Based on our literature search, there are very few studies on automatic tip-burn detection specifically for indoor farms. Shimamura et al. (2019) introduced tip-burn identification in plant factories using GoogLeNet for two classifications of tip-burn types from single lettuce images [7]. The images were collected under white lighting and a white background. Instead of single plant images, Gozzovelli et al. (2021) conducted tip-burn detection based on images of very dense plant canopies in plant factories, and Wasserstein generative adversarial network (WGAN) was applied to solve the problem of dataset imbalance (between healthy and unhealthy lettuces). YOLOv2 backbone Darknet-19 was used to detect tip-burns in their study [36]. Most recently, Franchetti and Pirri (2022) developed a new method for tip-burn stress detection and localization that was also deployed on plant canopy images. This technique used classification and self-supervised segmentation to locate and closely segment the stressed regions using ImageNet-1000 backbone Resnet-50V2 [37]. All of the studies collected the data on the uniform setup background, especially lighting conditions [7,36,37]. 

## 3. Materials and Methods

### 3.1. Plant Material and Cultivation Condition

Due to the limited availability of a public dataset of tip-burn lettuce grown in an indoor environment, to acquire the dataset, the lettuce plants were grown under conditions that can manifest tip-burns. As mentioned before, several conditions contribute to tip-burn in plants cultivated on indoor farms, including high temperature, high light intensity, extended day length, poor air flow, and a high concentration of nutrient solution [1,3,4,7]. Therefore, in this experiment, the aforementioned considerations were taken into account while estimating and adjusting the parameters for growing tip-burn lettuce.

The experiments were conducted in a small-scale cultivation room located at the Bioproduction and Machinery Laboratory, Tsukuba-Plant Innovation Research Center (T-PIRC), University of Tsukuba, Japan, in the spring season during the period from March to June 2022. Green leaf lettuce seeds (*Lactuca sativa*) (Sakata Seed Corporation, Yokohama, Japan) were sown in hydroponic sponges for 14 days before they were transplanted to the hydroponic setup based on the nutrient film technique (NFT). Two sets of hydroponic systems were constructed from food-grade polyvinyl chloride (PVC) pipes, with each set consisting of three layers. The hydroponic systems were occupied with 189 total lettuce plants. The growing cycle was repeated twice. The lettuce plants were cultivated under a fully artificial light source with a combination of red, blue, and white LEDs, which contained wavelengths that are suitable for the plant photosynthesis process. 

The lettuce cultivated in a solution culture had its roots immersed in a hydroponic nutrient solution (Hyponica Liquid Fertilizer, Kyowa Co., Ltd., Takatsuki, Japan). Initially, all the lettuce plants were cultivated normally with the standardized nutrient solution for the first two weeks after they were transplanted into the system. Then, the deficient solutions were induced through the system through a supply of nutrient solutions that had higher concentrations of nitrogen but were deficient in calcium. To obtain faster symptoms of tip-burn lettuce, the nutrient pH and EC were also adjusted within the range between 6.2–7 and 2.0–2.5 mS/cm, respectively, every three days to make the nutrient parameters fluctuate. The photoperiod was set to 24 h/day during the vegetative stage and 20 h/day during the harvest stage. The temperature and humidity observed throughout the growing period were 18–23 °C and 48–56%, respectively. The experiment was carried out until the plants showed symptoms of tip-burn. 

### 3.2. Data Collection

**Figure 1:**
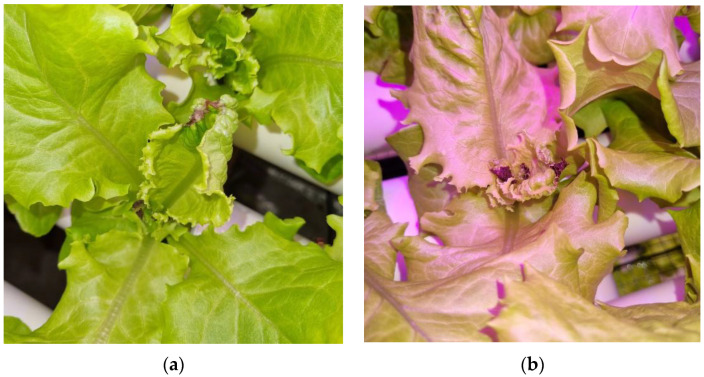
Example of captured images of tip-burn lettuce: (**a**) under white LEDs; (**b**) under red/blue LEDs.

Images of lettuce plants with tip-burn spots were collected starting from the first day they were visible by eye observation until the harvesting period. As tip-burn manifests on the tip of the leaves, the images were captured from the top of each infected plant with different angles, LED colors, and distances. The images were taken using a smartphone camera (Samsung Galaxy A50, Samsung Electronics Co., Ltd., Suwon, Korea) with aspect ratios of 1:1 and 3:4. Each image is 1080 × 2340 pixels. The total initial collection dataset of tip-burn lettuces was 538 images. The discolored leaf tips, brownish or blackish, indicated tip-burn (Figure 1).

### 3.3. Data Preparation

#### 3.3.1. Data Labeling

Before labeling, we resized all the images to a uniform size and constant resolution of 640 × 640. The resized images were then uniformly numbered. After that, we performed the labeling process using an open-source and free image annotation tool called LabelImg based on Python and Qt. Every visible and clear tip-burn spot was manually labeled by a rectangular bounding-box. Each output training image had a corresponding .txt file, containing the object class and coordinates of the bounding box of the upper left and lower right corners for each labeled tip-burn spot. In this experiment, tip-burn spots that were too small or highly indistinct were ignored and not labeled to prevent the possibility of these samples from degrading the neural network detection performance. The .txt file dataset was used for training in YOLOv4 and YOLOv5. Meanwhile, for CenterNet, we converted the .txt dataset into JSON format. 

#### 3.3.2. Data Augmentation and Splitting

**Figure 2:**
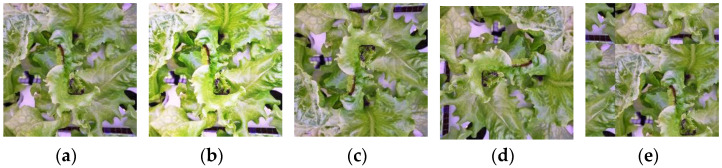
The data augmentation performed in this experiment: (**a**) original image; (**b**) increase image brightness; (**c**) flipped image vertically; (**d**) rotated image 90° clockwise; (**e**) shifted image horizontally and vertically.

A large dataset is required when training using a deep-learning algorithm as the model must extract and learn features from the images to identify and localize the targeted attributes. However, collecting a large dataset is very challenging and time consuming. Therefore, a data augmentation approach was performed to enhance our minimal dataset with the aim of being sufficiently able to be used to develop a reliable detection model [38]. In the experiment, several types of data augmentation were randomly used to increase the tip-burn images dataset. The data augmentations included brightness changes, image flips (mirrored original image horizontally or vertically), image rotations (rotated original image at 90° clockwise or counterclockwise), and shift (shifted original image horizontally and vertically and wrapped image around by the same image) (Figure 2).

The final dataset was expanded to a total of 2333 images as a result of the augmentation process. Table 1 presents the number of images that were split into training, validation, and test datasets. 

### 3.4. Training Process

#### 3.4.1. CenterNet

**Figure 3:**
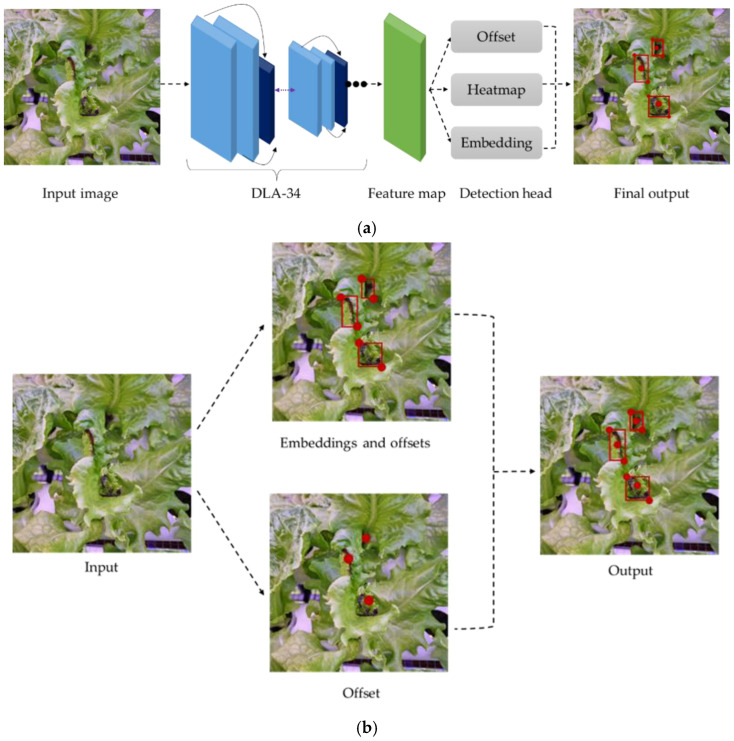
Working principle of CenterNet model (**a**) detection process of CenterNet based on backbone DLA-34; (**b**) tip-burn detection based on triplet key points.

CenterNet is an anchor-free target detection network which is an improvised model of CornerNet. The CenterNet-improved working principle makes them faster and more accurate than CornerNet. CenterNet recognizes each target object as a triplet of key points instead of a pair, as in CornerNet, which produces better precision and recall. In CenterNet, cascade corner pooling and center pooling were developed to enhance the data gathered from both the top-left and bottom-right corners and of an object to identify data from the targeted areas more clearly. The architecture of CenterNet begins with the input image entering the convolution neural network and employing cascade corner pooling to generate corner heatmaps, and center pooling to generate center heatmaps to the center point (heatmap), offset and boxes with three branches for prediction, to obtain the results. Then, a pair of detected corners and corresponding embeddings were utilized, as in CornerNet, to predict a potential bounding box. Finally, the final bounding boxes were identified by using the detected center key points [19] (Figure 3).

#### 3.4.2. YOLOv4 and YOLOv5

**Figure 4:**
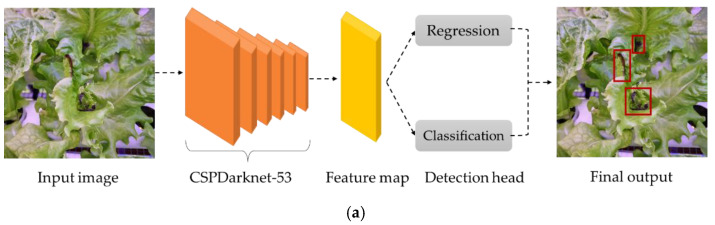

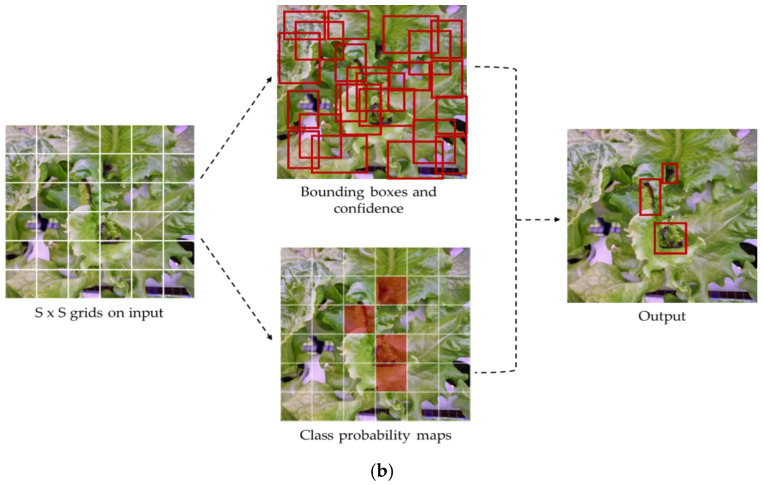
Working principle of the YOLO model: (**a**) detection process of YOLO model based on backbone CSPDarknet-53; (**b**) tip-burn detection based on bounding boxes.

The YOLO model is basically an object detector based on bounding boxes. During the detection process, the input image is segmented uniformly into equal grids. If the target is within the grid, the model generates a predicted bounding box and an associated confidence score. Then, the target for a particular object class is recognized when the center of the target-class ground truth lies within a specific grid (Figure 4). YOLOv4 is an improved model based on the backbone of YOLOv3, namely Darknet-53, to improve the accuracy of detecting small objects. A residual block skip connection and upsampling were included in Darknet-53, which greatly enhanced the algorithm’s accuracy. YOLOv4 further updates the network structure from YOLOv3 by changing to Cross Stage Partial Darknet-53 (CSPDarknet-53), where it utilizes convolution in the output layer. YOLOv4 additionally comes with batch normalization, a high-resolution classifier, and other tuning parameters to improve the detection result. The YOLOv4 model also employs multiscale prediction for detecting the final target, resulting in a better result in detecting small targets with high accuracy and speed. Additionally, YOLOv4 also offers a bag of freebies and a bag of specials to increase the performance of the algorithm. The bag of freebies includes complete intersection over union (CIoU) and is mostly related to the different data augmentations, including mosaic and self-adversarial training (SAT). The main purpose of SAT is to identify the region in the image that the network depends on the most during training and then modify the image to disguise this dependency. This teaches the network to generalize to other new features when finding the target class. Bags of specials, on the other hand, consist of distance IoU- non-maximum suppression (DIoU-NMS) and the additional activation function, mish activation. The working architecture of YOLOv4 starts with inputting images into the CSPDarknet-53 for feature extraction; then, it is sent to path aggregation network (PANnet) to extract information in layers near the input by transmitting features to the detector from multiple backbone levels [39]. 

YOLOv5 is a lighter version of the previous YOLO models that employs the PyTorch framework rather than the Darknet framework. It also uses CSPDarknet-53 as the backbone, similar to YOLOv4. The main difference in YOLOv5 is that it replaces the first three layers in the backbone of the YOLOv3 algorithm with a focus layer. The aim of the focus layer is to minimize the model size by eliminating certain parameter layers and parameters, reducing floating point operations per second (FLOPS) and CUDA memory, and increase the forward and backward speeds while minimizing the impact on the mean average precision (mAP) [40]. This process speeds up inference speed, improves accuracy, and reduces the computational load. In addition, YOLOv5 also utilizes PANet as its head part. The most significant improvements from YOLOv5 are auto-learning bounding box anchoring.

Training of the YOLOv4 and CenterNet models was conducted on a desktop PC with an NVIDIA^®^ GTX 1650™ with 4 GB GPU and an Intel^®^ Xeon™ E5-1607 CPU with 32 GB of RAM memory. The YOLOv5 model was trained on the cloud platform known as Google Colaboratory, a web-integrated development environment (IDE) with the GPU of Tesla P100-PCIE 16GB. 

### 3.5. Performance Metrics 

In our dataset, the target for the deep-learning models was to detect the tip-burn spot, hence we only had a single class, labelled as tip-burn. Outside of the target area or bounding box was predicted as background. Several performance metrics were used to evaluate the performance of the model, including intersection over union (IoU), precision, recall, and mean average precision (mAP). These metrics are based on PASCAL VOC which are well-known for use in object detection [41]. IoU is a measure of the distance between the predicted box and the ground truth box which ranges from zero (no overlap) to one (complete overlap). It highlights the preciseness of the algorithm in detecting the tip-burn. With the IoU, the total number of true-positives (TP), false-positives (FP), and false-negatives (FN) were determined. In this experiment, a TP is a tip-burn spot that was detected as a tip-burn. A FP is when another object, i.e., background, was detected as a tip-burn, while FN is when the tip-burn spot is not detected. Precision and recall are the two metrics that are commonly used to evaluate objection models. Precision determines the accuracy and preciseness of TP detection (Equation (1)), whereas recall indicates the effectiveness of the trained model in identifying all the TPs (Equation (2)). The mAP is the area under the precision and recall curve (Equation (3)).

(1) $$\mathrm{Precision}=\frac{\mathrm{TP}}{\mathrm{TP}+\mathrm{FP}}$$

(2) $$\mathrm{Recall}=\frac{\mathrm{TP}}{\mathrm{TP}+\mathrm{FN}}$$

(3) $$\mathrm{mAP}=\frac{1}{C}\overset{T}{\underset{k=1}{\sum }}P(k)\Delta R(k)$$

where C is total class numbers, T is IoU threshold numbers, k is the IoU threshold, P(k) is the precision, and R(k) is the recall. 

Loss function in object detection is often used to indicate the degree of discrepancy between the predicted value and the true value of the model. There are three main losses in YOLOv5 denoted as: bounding box loss (box_loss), objectness loss (obj_loss), and classification loss (cls_loss). Box_loss represents bounding box regression loss to evaluate the preciseness of predicted bounding box on the target object (Equation (4)). Obj_loss is an objectness loss used to measure a confidence that the object falls in the proposed region of interest (Equation (5)). Cls_loss is a classification loss (Equation (6)).

(4) $${\mathrm{Box}\_\mathrm{loss}=\lambda }_{\mathrm{coord}}\overset{S^{2}}{\underset{i=0}{\sum }}\overset{B}{\underset{j=0}{\sum }}I_{i,j}^{\mathrm{obj}}{\mathrm{bj}(2\;-\;w}_{i}{\;\times \;h}_{i}) [{\left(x_{i}\;\times \;{\hat{x_{i}}}^{j}\right)}^{2}+{\left(y_{i}\;\times \;{\hat{y_{i}}}^{j}\right)}^{2}+{\left(w_{i}\;\times \;{\hat{w_{i}}}^{j}\right)}^{2}+{\left(h_{i}\;\times \;{\hat{h_{i}}}^{j}\right)}^{2}]$$

(5) $${\mathrm{Obj}\_\mathrm{loss}=\lambda }_{\mathrm{noobj}}\overset{S^{2}}{\underset{i=0}{\sum }}\overset{B}{\underset{j=0}{\sum }}I_{i,j}^{\mathrm{noobj}}{\left(c_{i}\;-\;\hat{c_{j}}\right)}^{2}{+\lambda }_{\mathrm{obj}}\overset{S^{2}}{\underset{i=0}{\sum }}\overset{B}{\underset{j=0}{\sum }}I_{i,j}^{\mathrm{obj}}{\left(c_{i}\;-\;\hat{c_{j}}\right)}^{2}$$

(6) $${\mathrm{Cls}\_\mathrm{loss}=\lambda }_{\mathrm{class}}\overset{S^{2}}{\underset{i=0}{\sum }}\overset{B}{\underset{j=0}{\sum }}I_{i,j}^{\mathrm{obj}}\underset{C\epsilon \mathrm{classes}}{\sum }p_{i}\left(c\right)\log(\hat{p_{l}}\left(c)\right)$$

where s^2^ is the grids number, B is bounding boxes in each grid, $\lambda_{\mathrm{coord}}$ is the coefficient of position loss, $\hat{x}$ and $\hat{y}$ denote the target true central position, $\hat{w}$ is the target width, and $\hat{h}$ is the target height. $\lambda_{\mathrm{noobj}}$ is the coefficient of no object existing in the bounding boxes. $c_{i}$ and $\hat{c_{j}}$ represent the true confidence of bounding box and predicted confidence of bounding box, respectively. $\lambda_{\mathrm{class}}$ is the coefficient of classes loss. $p_{i}\left(c\right)$ is defined as the class probability of the target, and $\hat{p_{l}}\left(c\right)$ is the true value of the class.

Therefore, the total loss function is accumulated by using Equations (4) to (6) and expressed as:

(7) Loss=Box_loss+Obj_loss+Cls_loss

## 4. Results

### 4.1. Training

The total number of datasets in this study was 2333, from which 1750 images were used for training, 433 images for validation, and the remaining 150 images for testing. The training settings for the three models were applied differently according to the model and dataset suitability (Table 2).

Table 3 shows the comparison of the validation results at 50% IoU for the three models. CenterNet shows a recall value of 58% with mAP of 81.2%. The YOLOv5 model yielded the highest training accuracy, with mAP of 84.1% and a recall score of 79.4%. The mAP value at the same IoU level for YOLOv4 was 76.2%, which was the lowest among all the models. Therefore, the YOLOv5 model had a relatively better performance in training than both the CenterNet and YOLOv4 models.

**Figure 5:**
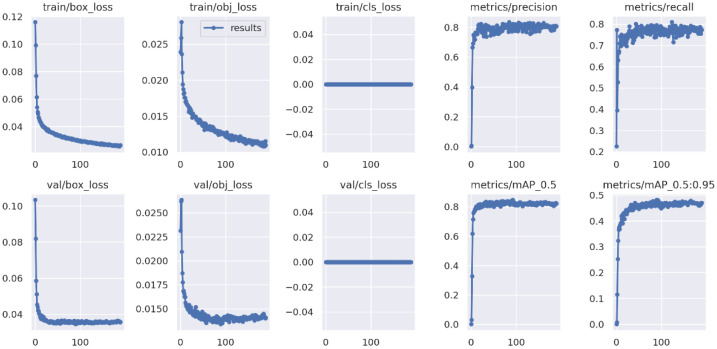
Training and validation results obtained from YOLOv5.

Figure 5 shows the results of losses and metrics obtained from the training and validation process using YOLOv5. Initially, the losses showed a rapid decline when reached at around 89 epochs from a total of 200 epochs. In this study, the classification loss was constant at 0 as we only trained for a single class, tip-burn. At the same time, as the losses decreased, the precision, recall, and mAP continued to increase before they reached the plateau phase. The training was stopped early at 189 epochs as no further improvement was observed (Figure 5).

### 4.2. Testing for Detection Accuracy

It is important to precisely detect tip-burn spots on lettuce to develop an effective automated tip-burn detection method for lettuce grown indoors. To evaluate the detection model, we tested 150 images by using the best trained weight obtained from each model. From the testing results, YOLOv5 shows the highest accuracy at 82.8% followed by CenterNet at 78.1%. YOLOv4 gives the lowest detection accuracy of only 67.6% (Table 4). The results indicate that the YOLOv5 model demonstrates better detection accuracy compared with CenterNet and YOLOv4.

**Figure 6:**
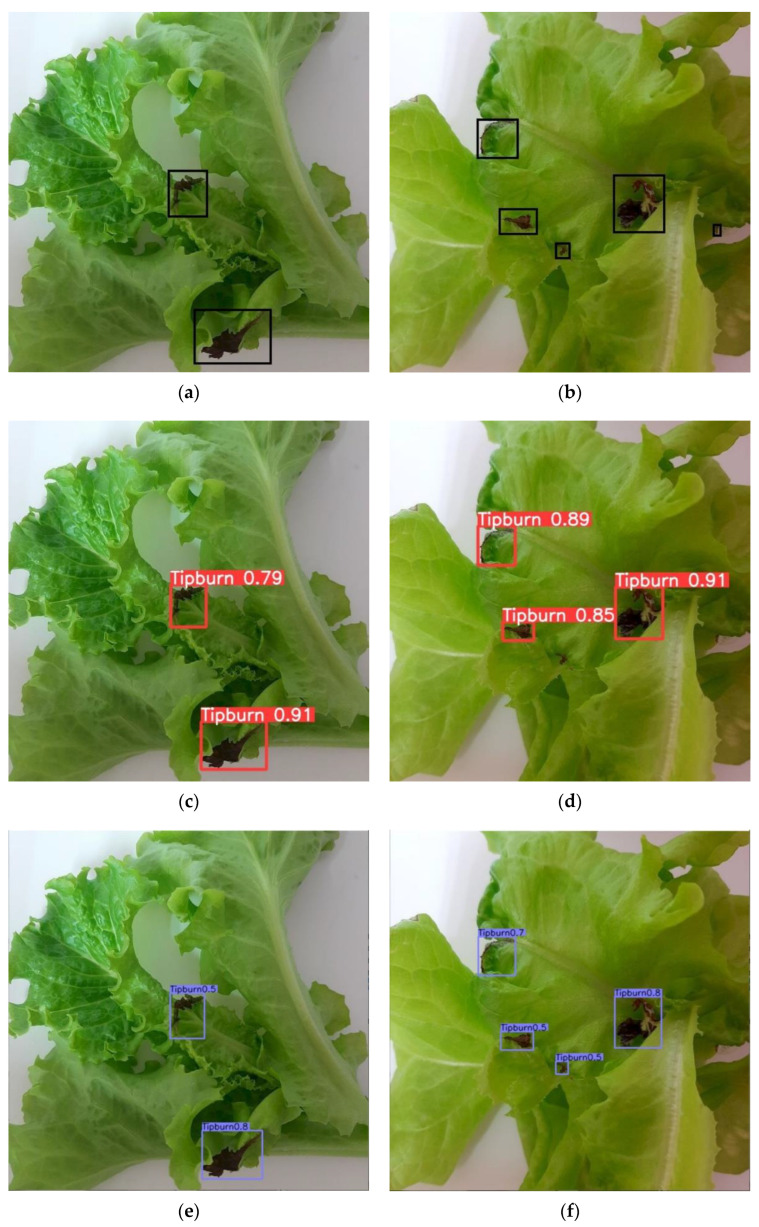

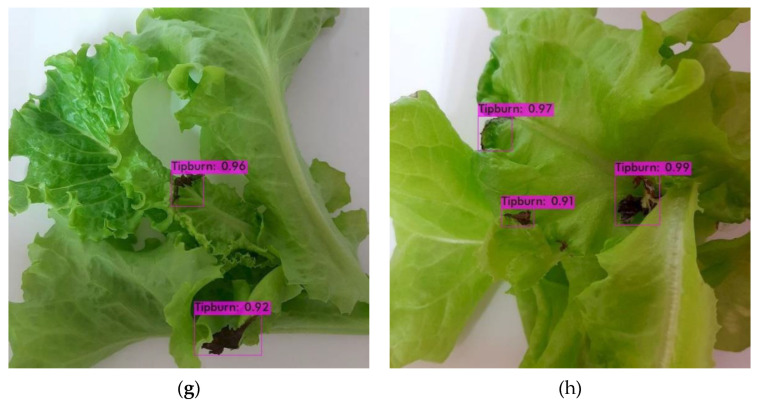
Detection of tip-burn under white light conditions from (**a**,**b**) manually labelled; (**c**,**d**) YOLOv5; (**e**,**f**) CenterNet; (**g**,**h**) YOLOv4.

Figure 6 shows examples of tip-burn detection based on an image acquired under white LEDs. All models were able to detect all the tip-burn spots on the lettuce under white light conditions (Figure 6). However, YOLOv5 (Figure 6d) and YOLOv4 (Figure 6h) had misdetection of two tip-burn spots, whereas CenterNet only missed one spot (Figure 6f). This misdetection was plausible given that the very small tip-burn spots made it challenging for the models to detect.

**Figure 7:**
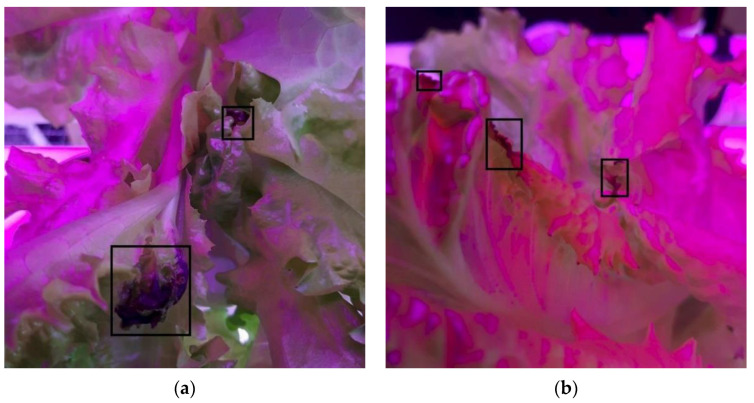

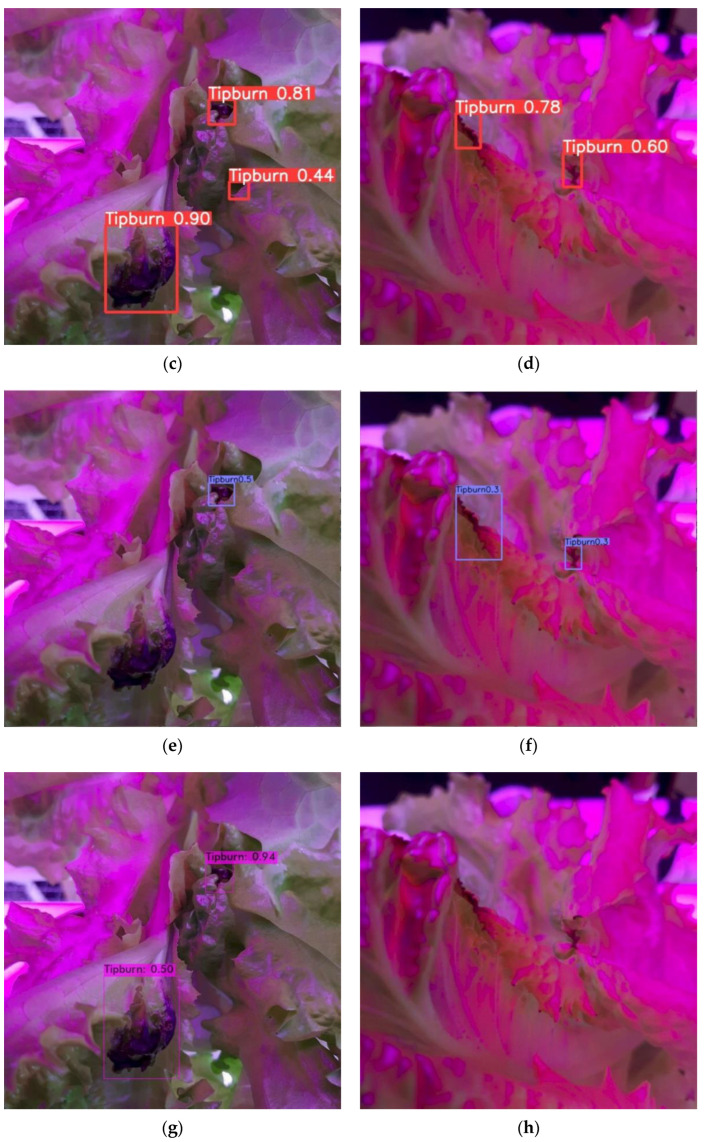
Detection of tip-burn under red/blue light conditions from (**a**,**b**) manually labelled; (**c**,**d**) YOLOv5; (**e**,**f**) CenterNet; (**g**,**h**) YOLOv4.

Detection under red/blue light conditions is shown in Figure 7. The YOLOv5 had false positives that falsely detected tip-burns that were not in the image (Figure 7c). CenterNet had one misdetection (Figure 7e). On the other hand, both YOLOv5 (Figure 7d) and CenterNet (Figure 7f) missed the detection of one tip-burn. Meanwhile, YOLOv4 struggled to detect all the tip-burn spots accurately in Figure 7h, where none were detected. Some of these misdetections occurred due to an overlapping between the leaves in the background of the exact tip-burn spot. Moreover, this is understandable under this light condition; it is very difficult for even a human to identify where the exact tip-burns are in the image.

**Figure 8:**
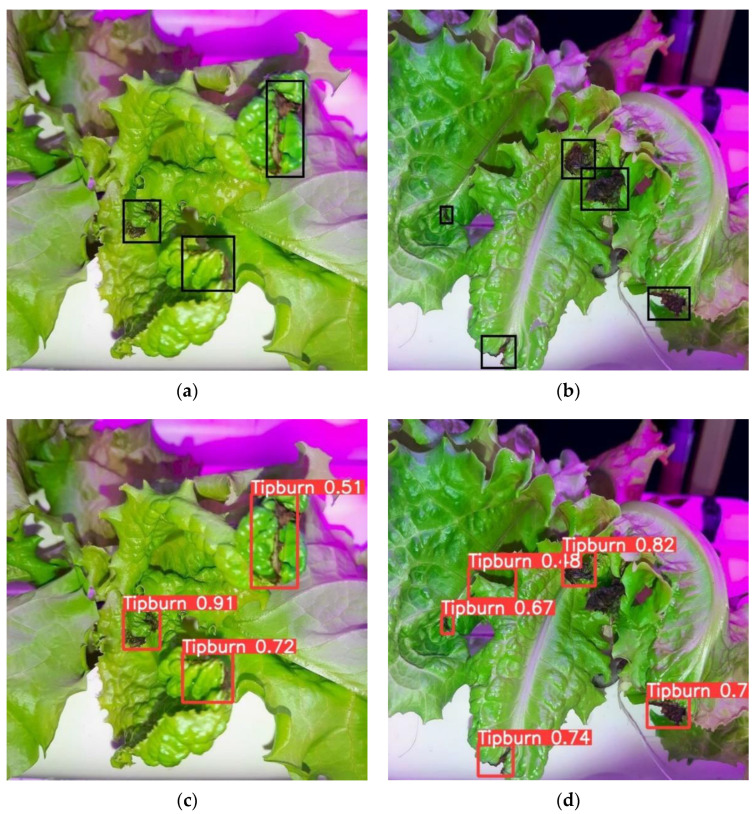

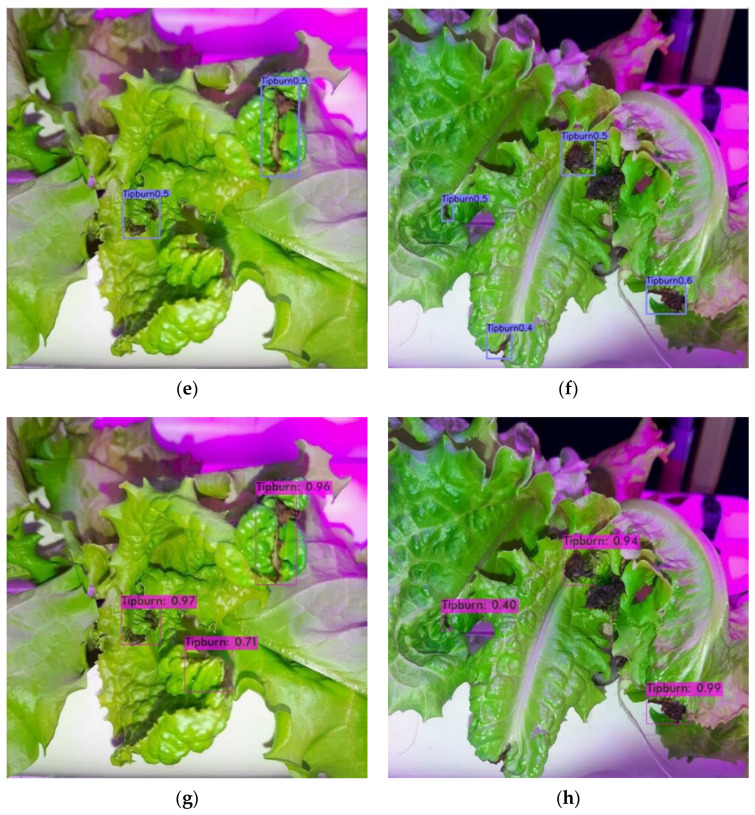
Detection of tip-burn under white/red/blue light conditions from (**a**,**b**) manually labelled; (**c**,**d**) YOLOv5; (**e**,**f**) CenterNet; (**g**,**h**) YOLOv4.

Figure 8 shows tip-burn detection under combination of white, red, and blue LEDs. The total of TP from the two proposed test images were eight (Figure 8a,b). Both YOLOv5 (Figure 8c) and YOLOv4 (Figure 8g) were able to detect all the tip-burn spots accurately, while CenterNet missed one (Figure 8e). YOLOv5 had a FP and one misdetection (Figure 8d). CenterNet had only one misdetection (Figure 8f), whereas YOLOv4 had two missed spots (Figure 8h).

## 5. Discussion

Plant stress and disease detection is important not only for plants grown in the outdoor environment, but also for plants grown indoors. Although the main advantage of growing plants indoors is the potential to promote rapid growth for fast returns, it also has drawbacks, particularly the occurrence of tip-burn leaves [1]. In this paper, the detection of tip-burn lettuce grown on an indoor farm using three different deep-learning models based on a one-stage detector was performed. The models used in the study were CenterNet, YOLOv4, and YOLOv5. Overall, YOLOv5 outperformed the other two tested models with the highest mAP of 82.8%. 

However, for tip-burn detection between different light conditions, most of the models could not perform well under red/blue LEDs. One of the reasons is due to the complex scenes, especially under red and blue lights, where the color of the tip-burn is difficult to differentiate from the background leaves, eventually confusing the models and producing a misdetection. Additionally, as mentioned before, it is very difficult for even a human to see and detect the tip-burn under these light conditions. In this case, a thermal camera may be useful for collecting a dataset under different light conditions, as it is not affected by visible light [42]. Apart from that, there may also be some errors and inconsistencies during the labeling process with certain tip-burn locations not being labeled properly. 

Datasets are the most important part of every deep-learning algorithm. The quantity and quality of the input data are very crucial to generate the best model and most efficient system. In this paper, the dataset used for the training is relatively small compared with other deep-learning datasets. In this experiment, we prepared all the tip-burn datasets from several batches of plant growth, starting from seeds’ germination, seedling, and growing in an indoor environment. It is very difficult and time consuming to generate the tip-burn dataset from batches after batches in the indoor plant growing systems. Moreover, the generation of the tip-burn is also randomly occurring where the deficiency is observed. Furthermore, the training dataset contained more images of tip-burn lettuce under white light than under the other light conditions. The imbalanced dataset may have a bias toward white light conditions during training, causing the models to lack enough data to learn and resulting in the low detection of tip-burns under red/blue LED conditions. We believe that the detection accuracy may be improved by collecting and appropriately labeling more images, particularly by balancing the image numbers under red/blue light conditions. Since growing batch data collection is difficult, we recommend exploring advanced data augmentation strategies, such as generative adversarial network (GAN), to produce additional high-quality artificial datasets. The GAN method can produce artificial images realistically as brand-new data compared with traditional augmentation methods such as flipping or rotating [36]. On the other hand, instead of using well-known performance metrics, it is also recommended to choose performance metrics based on dataset conditions either balanced or imbalanced dataset, which can provide more comprehensive perspective on the performance of the deep-learning models [43,44].

From this study, it is also noteworthy that a complex model with large parameter numbers, such as YOLOv4, had the lowest overall accuracy. YOLOv5 and CenterNet were both smaller and lighter than YOLOv4, but they could produce better accuracy. As stated in Table 3, we chose the YOLOv5s model and CenterNet ctdet_coco_dla_2x model, which are both smaller version of their original models. This indicates that when the target class and dataset are minimal, employing a model with large parameters may not be effective and suitable. Therefore, utilizing a comparatively small model or network makes it still possible to achieve accurate results with less computing facilities to develop a commercial system for detection of tip-burn lettuces. The capability of this small model of YOLOv5 can be further utilized for real-time monitoring of tip-burn lettuce detection in indoor farms, allowing for prompt and accurate interventions for early detection of tip-burn, such as modifying the growing environment with suitable humidity, temperature, light settings, and air movement, and additionally, providing calcium nutrients to the plants in the indoor farming systems.

## 6. Conclusions

Lettuce plants cultivated in indoor environments are fully reliant on artificial lighting sources. The ability to modify growing environments and lighting conditions can help to accelerate plant growth. However, this rapid growth process prevents the developing leaves from receiving an adequate amount of calcium, hence intensifying the incidence of tip-burn on plants grown indoors. Therefore, in this study, a method for the detection of tip-burn lettuce cultivated in an indoor environment under different light conditions was developed. Images of tip-burn lettuces were collected under white, red, and blue LEDs and were used as training, validation, and testing datasets for a deep-learning detection method. The detection method used was based on one-stage detectors, namely, CenterNet, YOLOv4, and YOLOv5. Among the three tested models, YOLOv5 achieved the best accuracy with 84.1% mAP. Nevertheless, further improvements can be made by using a larger dataset with balanced conditions to increase the detection accuracy. We believe this study provides an additional foundation for the automation of plant disease or stress detection in indoor farming systems, particularly under the different growing light conditions. This work can be extended in the future by employing this model for real-time application.

## Figures and Tables

**Table 1:** Data splitting for training.

Model	Train	Validation	Test
CenterNet	1750	433	150
YOLOv4-5	1750	433	150

**Table 2:** Configuration for training for selected deep learning algorithms.

Model		Input Size	Learning Rate	Batch Size	Epoch
CenterNet	ctdet_coco_dla_2x	416 × 416	0.00025	24	30
YOLOv4	-	416 × 416	0.0001	16	4000
YOLOv5	YOLOv5s	416 × 416	0.01	32	200

**Table 3:** Comparison of recall and mAP between CenterNet, YOLOv4, and YOLOv5 deep-learning algorithms.

Model	Recall (%)	mAP (%)
CenterNet	58.0	81.2
YOLOv4	74.0	76.2
YOLOv5	79.4	84.1

**Table 4:** Comparison detection of mAP between CenterNet, YOLOv4, and YOLOv5 deep-learning algorithms.

Model	mAP (%)
CenterNet	78.1
YOLOv4	67.6
YOLOv5	82.8

## Data Availability

The dataset that was generated and analyzed during this study is available from the corresponding author upon reasonable request, but restrictions apply to the data reproducibility and commercially confident details.
